# Supplementary material for: Dynamic representation of sound locations during task engagement in marmoset auditory cortex
Source: bioRxiv. 2025 Aug 19:2025.08.14.669832. Preprint. [Version 1] doi: 10.1101/2025.08.14.669832 (PMC12393296; doi:10.1101/2025.08.14.669832)
Supplement: Supplement 1 [file NIHPP2025.08.14.669832v1-supplement-1.pdf]

bioRxiv preprint doi: https://doi.org/10.1101/2025.08.14.669832; this version posted August 19, 2025. The copyright holder for this preprint (which was not certified by peer review) is the author/funder, who has granted bioRxiv a license to display the preprint in perpetuity. It is made available under aCC-BY 4.0 International license.

Supplementary Figures:

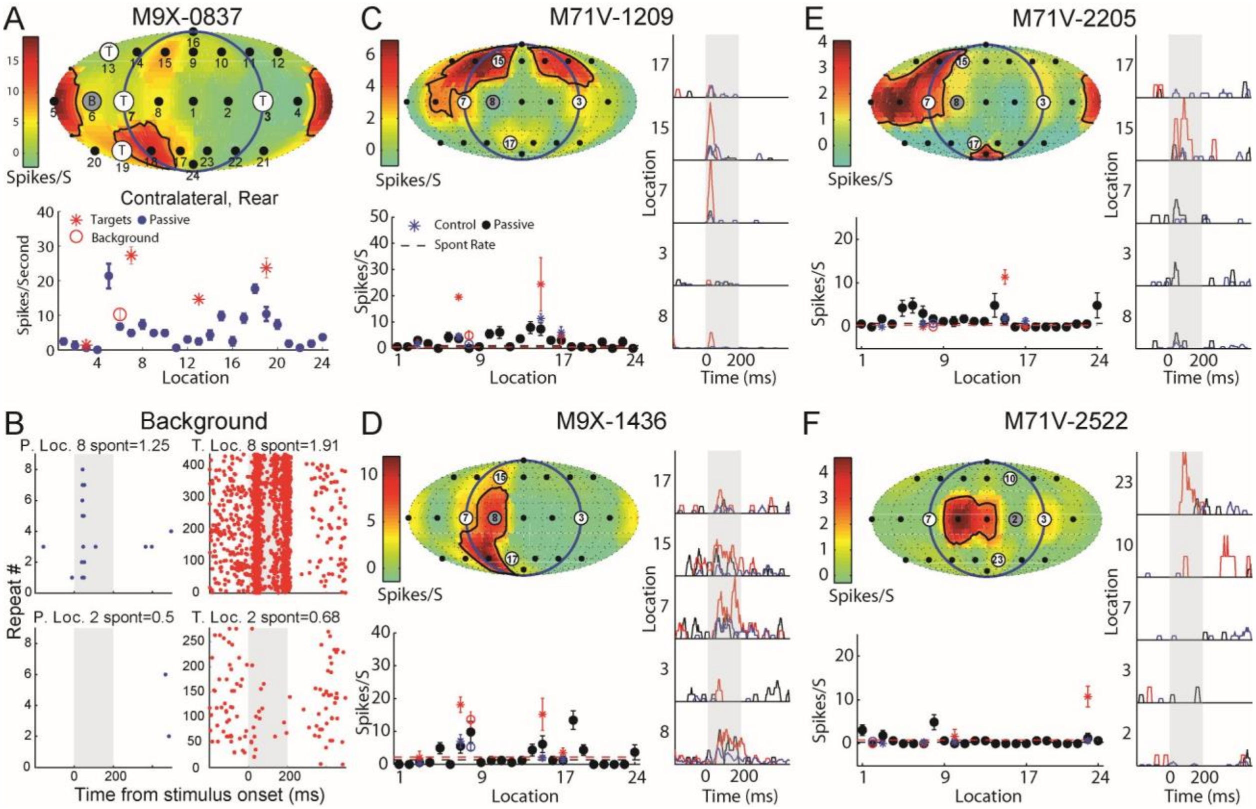

Supplementary Figure 1. More example units with firing rate increase outside the spatial RFs (related to Figure 2). (A) An example session (same unit in Fig. 2A) where the background location (#6) and two target locations (#13 and #19) were located at the rear of the animal. (B) Spike raster from two example units (Fig. 2A and 2F) at the background locations (#8 and #2) under passive (left) and target (right) conditions. Notice there were many more trials under the target locations. (C-F) Four more example units. Here, we used black, blue, and red colors to represent 24 locations during passive, control, and behaving conditions (five targets/background locations), respectively. Spontaneous firing rates were indicated with colored dashed lines.

bioRxiv preprint doi: https://doi.org/10.1101/2025.08.14.669832; this version posted August 19, 2025. The copyright holder for this preprint (which was not certified by peer review) is the author/funder, who has granted bioRxiv a license to display the preprint in perpetuity. It is made available under aCC-BY 4.0 International license.

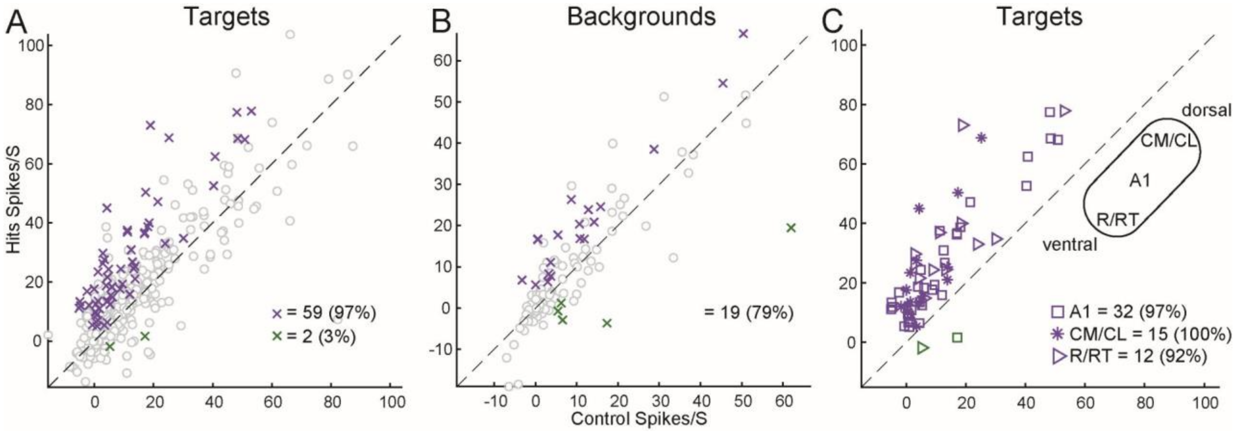

Supplementary Figure 2. Firing rate during the behavior and control conditions (related to Figure 3A-C). (A) Comparisons of firing rates in the hits (i.e., successful behavioral choice) and control conditions at all target locations. Gray circles represent the nonsignificantly modulated target locations (249 above and 199 below the diagonal). (B) Similar to (A) but at the background locations. There were 53 and 55 nonsignificant background locations above and below the diagonal, respectively. (C) Same data as the significantly increased (pink) and decreased (green) firing rate at target locations shown in (A), but with three different shapes (asterisk, square, and right-pointing triangle) to distinguish the three auditory cortical areas (A1, CM/CL, and R/RT).

bioRxiv preprint doi: https://doi.org/10.1101/2025.08.14.669832; this version posted August 19, 2025. The copyright holder for this preprint (which was not certified by peer review) is the author/funder, who has granted bioRxiv a license to display the preprint in perpetuity. It is made available under aCC-BY 4.0 International license.

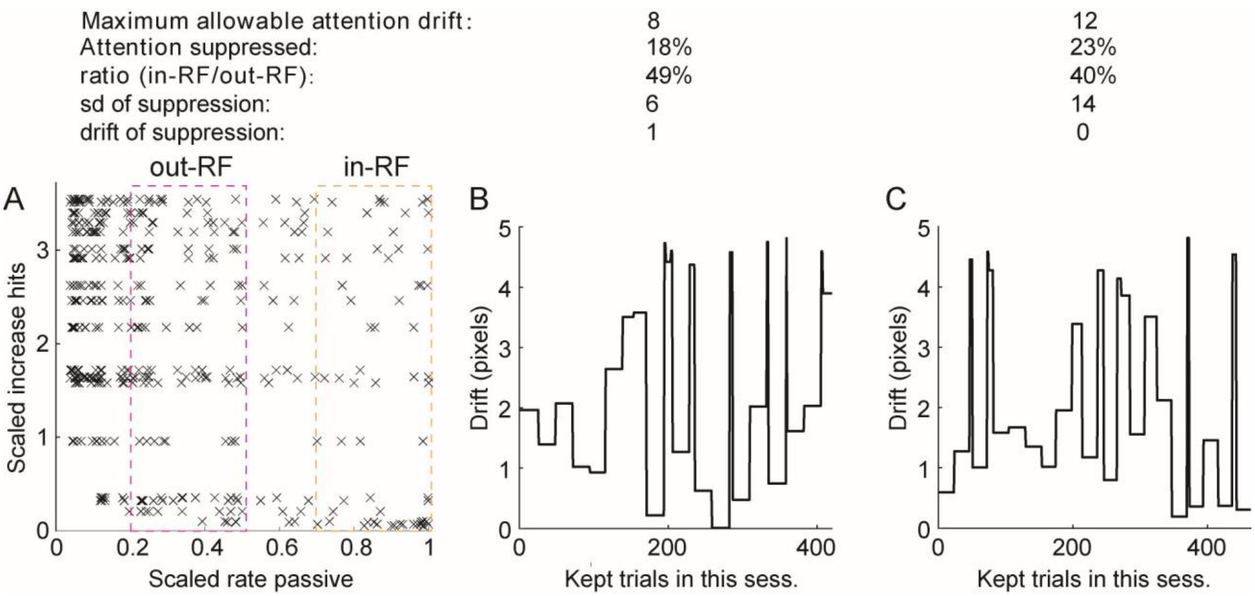

Supplementary Figure 3. Increased firing rates to target locations increased firing rates relative to background locations (related to Figure 4). (A) Firing rate during task plotted vs. passive firing rate for all data points that were larger than 1, both scaled by the maximum firing rate in the passive condition. The scaled passive rates between 0.2 to 0.5 and 0.7 to 1 were considered as outside and inside of receptive fields, respectively. (B-C) The drift of attention fields in all kept trials from two example sessions. The maximum drifts were only 5 pixels in the kept trials, although the allowable drifts were much larger.

bioRxiv preprint doi: https://doi.org/10.1101/2025.08.14.669832; this version posted August 19, 2025. The copyright holder for this preprint (which was not certified by peer review) is the author/funder, who has granted bioRxiv a license to display the preprint in perpetuity. It is made available under aCC-BY 4.0 International license.

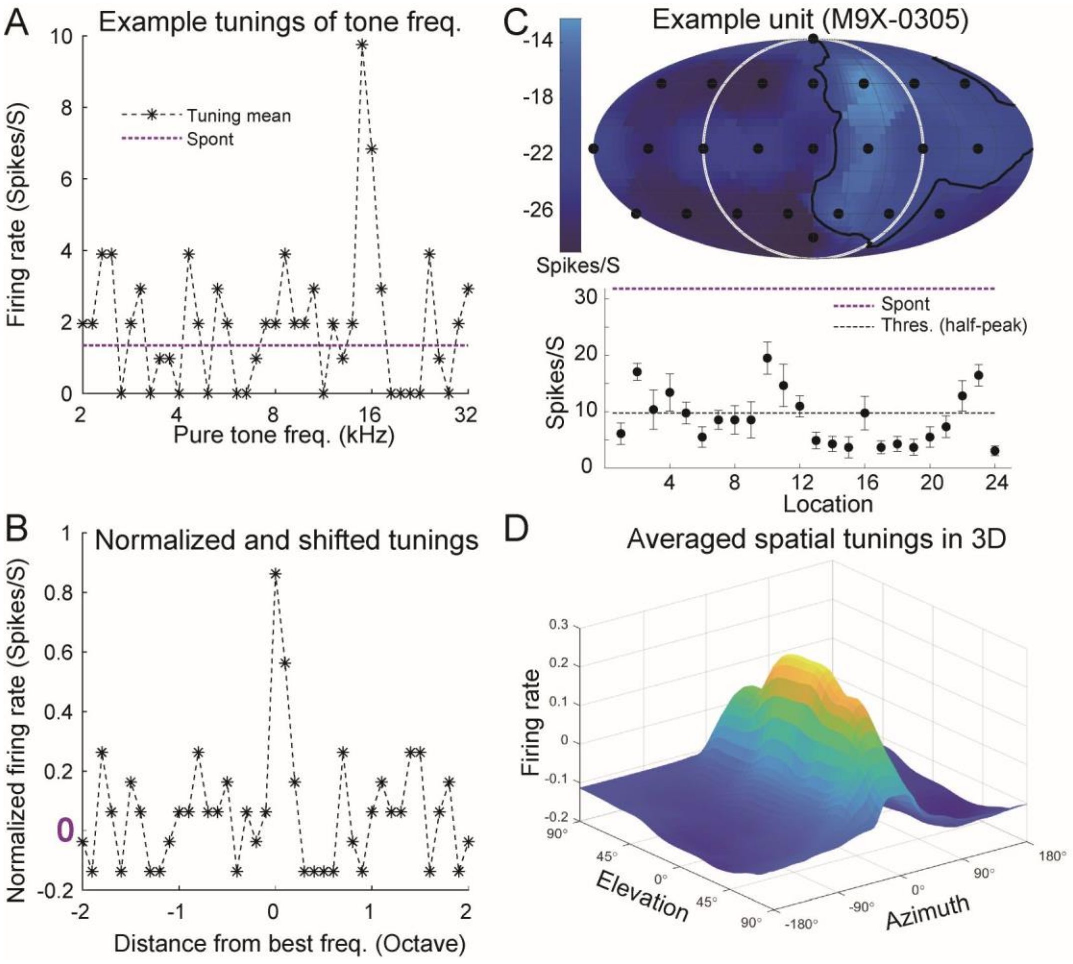

Supplementary Figure 4. Suppressed neural firing rate to the nonpreferred sound stimuli (related to Figure 5C). (A) Averaged neural firing rate to 31 sound frequencies (4 octaves, 8 stimuli per octave) of an example unit. (B) The tuning curve was normalized by the maximum firing rate at 16 kHz (the spontaneous firing rate equated to “0”). It was further circularly shifted so that there were 15 frequencies (2 octaves) at both the left and right sides of the peak firing rate. (C) An example unit that was suppressed at all 24 sound locations. Notice that the neuron was still significantly tuned to sound locations (ANOVA, p<0.01). (D) A 3D view of averaged spatial receptive fields with both color and height at the third axis represented the firing rate.

bioRxiv preprint doi: https://doi.org/10.1101/2025.08.14.669832; this version posted August 19, 2025. The copyright holder for this preprint (which was not certified by peer review) is the author/funder, who has granted bioRxiv a license to display the preprint in perpetuity. It is made available under aCC-BY 4.0 International license.

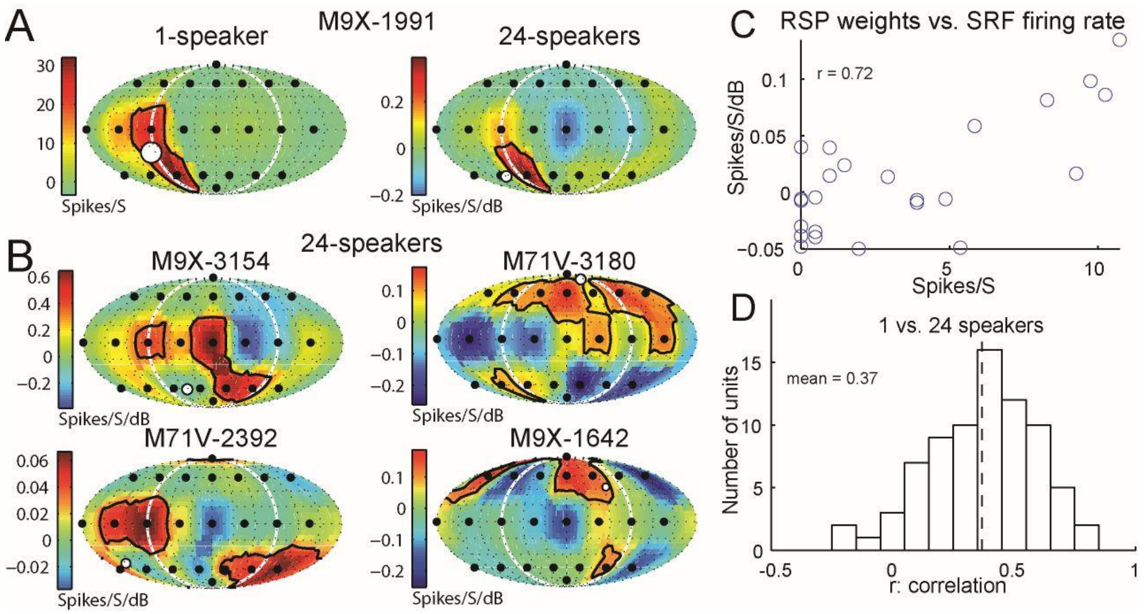

Supplementary Figure 5. Suppressed neural firing rate to the random spatial profile (RSP) sound stimuli (related to Figure 5D). (A) An example unit showed consistent spatial receptive fields under two stimulus paradigms. The position and size of white dots indicate the center of receptive fields and their tuning selectivity, respectively. (B) Four more example units all showed suppressed firing rates at more than one sound location using the RSP stimuli. (C) The scatter plot (X-axis: 1-speaker, Y-axis: 24-speakers) of neural activities at 24 sound locations under two stimulus paradigms. The correlation (r) of neural activities was 0.72. (D) The histogram of correlation for all 77 units.
